# Factors associated with anemia in chronic kidney disease in the Brazilian population: national health survey

**DOI:** 10.3389/fnut.2026.1714573

**Published:** 2026-02-09

**Authors:** Letícia Cristina Machado de Sousa, Nathalia Rabello Silva, Bianca Uliana Picolo, Marcus Vinícius de Pádua Netto, Luciana Saraiva da Silva

**Affiliations:** 1 Faculty of Medicine, Federal University of Uberlândia, Uberlândia, Minas Gerais, Brazil; 2 Prof. Luiz Ricardo Goulart Nanobiotechnology Laboratory, Institute of Biotechnology, Federal University of Uberlândia, Uberlândia, Minas Gerais, Brazil

**Keywords:** anemia, anemia of chronic disease, associated factors, chronic kidney disease, national health survey

## Abstract

**Introduction:**

Chronic kidney disease (CKD) is a public health issue often accompanied by anemia from its early stages. Identifying associated factors is essential, especially given the limited data on the Brazilian population and the outcomes of anemia among individuals with CKD.

**Objective:**

To investigate the factors associated with anemia in individuals with CKD in the Brazilian population.

**Methods:**

A cross-sectional study was conducted using the 2013 National Health Survey (PNS) laboratory database. Socioeconomic, demographic, and clinical variables (sex, age, education, race/color, nutritional status, arterial hypertension (AH), diabetes mellitus (DM), hypercholesterolemia, cardiovascular disease (CVD), smoking, and excessive alcohol consumption) were evaluated. Associations between anemia and the stages of CKD were examined using multivariable logistic regression models.

**Results:**

8,952 individuals were evaluated. The prevalence of anemia in the total population was 10.1%, and in the population with CKD, it was 15.1%. Anemia was found to be more prevalent among women, older adults, individuals with lower educational, and those of mixed and black race/color. In the fully adjusted model, women (OR 1.59; CI, 1.35–1.88), black individuals (OR 1.76; CI, 1.36–2.26), and those with AH (OR 1.26; CI, 1.04–1.52) or DM (OR 1.30; CI, 1.00–1.69) exhibited a higher likelihood of anemia. Conversely, individuals with higher educational levels (OR 0.65; CI, 0.49–0.87), smokers (OR 0.71; CI, 0.56–0.91) those with a eutrophic body status (OR 0.66; CI, 0.49–0.90), and obese individuals (OR 0.49; CI, 0.36–0.67) had lower likelihood of anemia compared to underweight individuals. Individuals with advanced stages of CKD had a higher likelihood of anemia compared with those in early stages, and this association remained significant after adjustment for sociodemographic and clinical variables, with the highest odds observed in stages 4 and 5.

**Conclusion:**

This study made it possible to identify the variables related to the presence of anemia in the different stages of CKD, such as AH, DM, sex, education, race/color, and nutritional status.

## 1 Introduction

Chronic kidney disease (CKD) is a public health problem that affects more than 800 million people worldwide (1), with a global prevalence of 11%−13% (2). In 2017, CKD ranked as the 12th leading cause of mortality, accounting for 1.2 million deaths (3). It is projected that between 2016 and 2040, it will rise to the fifth position among the leading causes of premature deaths (4).

According to the Brazilian Society of Nephrology, CKD is an illness unknown to many people, which is an aggravating fact, given that 10 million Brazilians have some degree of renal impairment (5). However, even in its early stages, CKD presents complications, such as anemia, mineral and bone disorders, metabolic acidosis, and malnutrition (6), which may increase in prevalence as the disease progresses (7).

In this sense, anemia is a common complication in these patients (8). In a study carried out with the United States population, a prevalence of 15.4% was found in individuals with CKD, with this prevalence increasing as the disease progressed (9, 10). According to *Kidney Disease: Improving Global Outcomes Anemia Working Group* (9), anemia is defined as serum hemoglobin (Hb) concentrations < 12 g/dl in women and < 13 g/dl in men. In CKD, anemia is multicausal, with the main triggering factors being the reduction in the production of erythropoietin–which is a hormone synthesized, primarily in the kidneys–in addition to resistance to this hormone, reduction in the half-life of red blood cells and deficiency of iron (11, 12). Also, factors such as body mass index (BMI), biochemical parameters (albumin and leukocytes), diabetic nephropathy, and CKD stage are associated with the development of anemia in patients with CKD (13).

Previous studies have demonstrated that sociodemographic issues, late screening, advanced stages of CKD, and lifestyle may also be associated with the increased prevalence of anemia in patients with CKD, as more vulnerable groups tend to have restricted access to health services, as well as different eating patterns (14–16).

Considering the scarcity of studies representative of the Brazilian population and the adverse outcomes of the presence of anemia in the CKD population, such as low quality of life, increased risk of developing cardiovascular disease and death (17, 18), it is important to identify the main associated factors in this population. Despite the high burden of CKD in Brazil, nationally representative studies combining laboratory-based CKD staging and anemia assessment remain scarce. Our study addresses this gap using a national laboratory subsample of the National Health Survey (PNS). Therefore, this study aimed to investigate the factors associated with anemia in individuals with CKD in the Brazilian population.

## 2 Methods

### 2.1 Study design and subjects

This cross-sectional study uses the laboratory database from the 2013 PNS. The laboratory test survey carried out in 2014 and 2015 is considered a second stage of the PNS. The PNS is a nationwide, household-based survey conducted by the Brazilian Institute of Geography and Statistics (IBGE) in partnership with the Ministry of Health (19).

The 2013 survey used a three-stage probability sample. The first stage consisted of census tracts (or sets of sectors), which formed the primary units; the second comprised households; and the third comprised adult residents aged 18 or over (20). Sixty thousand two hundred two individuals participated in the research and responded to individual interviews.

The selection of the subsample for the collection of biological material was planned in 25% of the census sectors, assuming a non-response rate of 20%; therefore, laboratory data from approximately 12 thousand individuals was expected. However, there were losses in the process of collecting laboratory tests due to the difficulty in locating the address by the contracted laboratory, the refusal of the selected resident to participate in the collection of biological material, the prolonged time between the initial application of the questionnaire and the visit of the laboratory agent, and the operational difficulties of transporting biological material. Therefore, the laboratory sample consisted of 8,952 people. To establish estimates for the Brazilian adult population, the study added post-stratification weights by sex, age group, race/color, and level of education according to major region (19).

### 2.2 Data collection and studied variables

The present study analyzed socioeconomic and demographic variables: sex (male and female); age categorized into age groups (18–29; 30–44; 45–59; 60 or more); education (no education/incomplete elementary school; complete elementary school/incomplete high school; complete high school/incomplete higher education; complete higher education) and race/color [white; black; brown and others (yellow and Indigenous)].

Furthermore, the following clinical variables were analyzed: nutritional status (underweight, eutrophy, and overweight), presence of arterial hypertension (AH), diabetes mellitus (DM), hypercholesterolemia, cardiovascular disease (CVD), and CKD. Information on AH, DM, hypercholesterolemia and CVD was based on self-report of previous medical diagnoses. Renal function was assessed using a single measurement of glomerular filtration rate (GFR) estimated from the Chronic Kidney Disease Epidemiology Collaboration (CKD-EPI) equation (21). According to Kidney Disease: Improving Global Outcomes (21), CKD can be classified into the following stages, according to the GFR: stage 1 (≥90 ml/min/1.73 m^2^); 2 (60–89.9 ml min/1.73 m^2^); 3a (45–59.9 ml min/1.73 m^2^); 3b (30–44.9 ml min/1.73 m^2^); 4 (15–29.9 ml min/1.73 m^2^) and 5 (< 15 ml min/1.73 m^2^).

Behavioral variables related to lifestyle were also considered, such as smoking and excessive alcohol consumption.

Concerning nutritional status, the cutoff point for underweight was considered to be a BMI < 18.5 kg/m^2^; for eutrophy, a BMI between 18.5 and 24.9 kg/m^2^ and for overweight, a BMI ≥ 25.0 kg/m^2^ for adults (22) and a BMI ≥ 27.0 kg/m^2^ for the elderly (23). For smoking, the following question was asked: “Do you currently smoke any tobacco products?” participants who answered “Yes, daily” and “Yes, less than daily” were considered smokers. Furthermore, to calculate excessive alcohol consumption, two questions from the PNS were considered: “How many days a week do you usually drink an alcoholic drink?” and “In general, on the day you drink, how many doses of alcoholic beverages do you consume?”, with one dose of alcoholic beverage equivalent to one can of beer, one glass of wine or one dose of cachaça, whiskey or any other distilled alcoholic beverage. Thus, excessive alcohol consumption was considered when the results of the two multiplied questions were equal to 15 or more drinks for men and eight or more drinks for women (24).

Anemia was defined as serum hemoglobin (Hb) concentrations < 12 g/dl in women and < 13 g/dl in men (9). Anemia was measured using a single hemoglobin measurement obtained from a complete blood count (CBC) laboratory test (20).

### 2.3 Statistical analysis

For the descriptive analysis, the following variables were considered according to anemia: sex, age group, education, race/color, nutritional status, AH, DM, hypercholesterolemia, cardiovascular disease, CKD, smoking, and excessive alcohol consumption. The prevalence of anemia was presented stratified by the CKD stage.

Qualitative variables were compared using the chi-square test, and quantitative variables using the *T*- student test. Regarding the association of anemia with CKD, the crude and adjusted models (for sociodemographic and clinical variables) were presented using logistic regression. Covariates were selected based on previous literature and biological plausibility regarding anemia and CKD (sociodemographic factors and comorbidities). We used a hierarchical approach: Model 1 adjusted for sociodemographic variables (sex, age group, education, race/color) and Model 2 additionally adjusted for clinical and behavioral variables (nutritional status, AH, DM, CVD, smoking, excessive alcohol consumption).

Missing data were evaluated for all study variables. Participants without laboratory measurements required to define the outcome (hemoglobin) and the exposure of interest (GFR) were not eligible for the analytical sample. For regression analyses, we performed a complete-case analysis, excluding observations with missing values in any covariate included in the adjusted models.

Statistical data were evaluated using Stata software (version 14.2). The significance level established was *p* ≤ 0.05.

### 2.4 Ethical aspects

The National Research Ethics Commission (CONEP) of the National Health Council (CNS) approved the PNS under opinion no. 3,529,376. Research participants signed the Free and Informed Consent Form.

## 3 Results

Eight thousand nine hundred fifty-two individuals were evaluated, and the prevalence of anemia in the total population was 10.1%, and in the population with CKD, it was 15.1%. In Table 1, the sociodemographic and clinical variables of the participants were presented according to anemia. It is noted that the majority of individuals were female (58.4%), of brown race/color (52.2%), aged between 30 and 44 years old (33%), and had incomplete elementary school (44.4 %). Higher prevalences of anemia were found in females (69.2%), aged 30–44 (32.4%), with low education (49.4%) and of brown race/color (53.5%). When comparing the same variable by the anemia, higher prevalences were found in individuals aged 60 or over (30.5%), with low education (49.4%) and black race/color (13.1%) and brown (53.5%).

**Table 1:** Characterization of the studied population by the presence or absence of anemia. PNS, 2014–2015.

**Characteristics**	**Variables**	**Total (%) CI 95%**	**Anemia**		***p*-value**
			**No (%) CI 95%**	**Yes (%) CI 95%**	
Sex	Male	41.6 (40.6–42.6)	42.8 (41.7–43.9)	30.8 (27.9–33.9)	< 0.001
	Female	58.4 (57.4–59.4)	57.2 (56.1–58.2)	69.2 (66.1–72.1)	
Age group	18–29 years	15.9 (15.1–16.6)	16.0 (15.2–16.8)	14.7 (12.5–17.1)	< 0.001
	30–44 years	33.0 (32.1–34.0)	33.1 (32.1–34.2)	32.4 (29.4–35.5)	
	45–59 years	27.7 (26.8–28.6)	23.3 (27.3–29.3)	22.4 (19.8–25.3)	
	60 years or more	23.4 (22.5–24.2)	22.5 (21.6–23.5)	30.5 (27.6–33.6)	
Education	No education/incomplete elementary school	44.4 (43.4–45.4)	43.8 (42.8–44.9)	49.4 (46.1–52.6)	0.003
	Complete elementary school/incomplete high school	15.5 (14.7–16.2)	15.5 (14.8–16.3)	14.7 (12.5–17.1)	
	Complete high school/incomplete higher education	29.9 (28.9–30.8)	30.0 (29.0–31.0)	28.4 (25.5–31.4)	
	Complete higher education	10.3 (9.6–10.9)	10.6 (9.9–11.2)	7.5 (6.0–9.4)	
Race/color	White	36.9 (35.9–37.9)	37.4 (36.4–38.5)	32.1 (29.2–35.3)	< 0.001
	Black	9.2 (8.6–9.8)	8.8 (8.2–9.4)	13.1 (11.1–15.5)	
	Others (indigenous and yellow)	1.7 (1.4–1.9)	1.7 (1.4–2.0)	1.2 (0.7–2.9)	
	Brown	52.2 (51.2–53.2)	52.1 (51.0–53.2)	53.5 (50.2–56.8)	
Nutritional status	Low weight	5.0 (4.5–5.4)	4.6 (4.1–5.1)	8.2 (6.5–10.1)	< 0.001
	Eutrophy	39.9 (38.9–40.9)	39.4 (38.4–40.5)	43.7 (40.5–47.0)	
	Overweight	55.1 (54.1–56.2)	55.9 (54.8–57.0)	48.0 (44.8–51.3)	
Arterial hypertension		24.3 (23.4–25.2)	23.5 (22.6–24.4)	31.0 (28.1–34.2)	< 0.001
Diabetes mellitus		7.6 (7.1–8.2)	7.2 (6.7–7.9)	11.1 (9.1–13.4)	< 0.001
Hypercholesterolemia		16.8 (16.0–17.7)	16.7 (15.8–17.6)	17.7 (15.2–20.5)	0.476
Cardiovascular disease		4.5 (4.1–4.9)	4.3 (3.9–4.7)	6.4 (5.0–8.2)	0.003
Smoking		14.5 (13.7–15.2)	14.7 (14.0–15.5)	11.9 (10.0–14.2)	0.019
Excessive alcohol consumption		28.8 (26.8–30.8)	28.3 (26.2–30.4)	34.4 (27.3–42.3)	0.107
CKD		10.5 (9.8–11.1)	10.0 (9.3–10.6)	15.1 (12.9–17.6)	< 0.001

In individuals with anemia, a higher prevalence of AH (31%), DM (11.1%), CVD (6.4%), and CKD (15.1%) and a lower prevalence of smoking (11.9%) were also identified. Other information can be seen in Table 1.

Table 2 shows the characterization of individuals with anemia stratified by CKD stage. It is observed that the presence of anemia was more significant in the more advanced stages of CKD, 52.6% and 42.1% in stages 4 and 5, respectively. Concomitantly, in the initial stages, the prevalence of anemia was lower, 11.0, 8.4, and 10.3% in stages 1, 2, and 3a, respectively.

**Table 2:** Distribution of anemia prevalence, according to CKD stages. PNS, 2014–2015.

**CKD stages**	**Anemia**		***p*-value**
	**Yes (%) CI 95%**	**No (%) CI 95%**	
1 (GFR ≥90 ml/min/1.73 m^2^)	11.0 (10.0–12.1)	89.0 (87.9–90.0)	**< 0.001**
2 (GFR 60–89.9 ml min/1.73 m^2^)	8.4 (7.6–9.3)	91.6 (90.7–92.4)	
3a (GFR 45–59.9 ml min/1.73 m^2^)	10.3 (8.3–12.7)	89.7 (87.3–91.7)	
3b (GFR 30–44.9 ml min/1.73 m^2^)	25.9 (19.8–33.0)	74.1 (67.0–80.2)	
4 (GFR 15–29.9 ml min/1.73 m^2^)	52.6 (30.6–73.7)	47.4 (26.3–69.4)	
5 (GFR < 15 ml min/1.73 m^2^)	42.1 (22.2–65.0)	57.9 (35.0–77.8)	

Bold values indicate statistically significant differences (*p* < 0.001).

In Table 3, the association of anemia stratified by CKD stages, individuals in more advanced stages of the disease were more likely to develop anemia than those in the initial stages, according to the crude model. The values were still significant after adjusting for sociodemographic variables (adjusted model 1) and sociodemographic and clinical variables (adjusted model 2), with odds ratios being 6.10 in stage 4 and 4.45 in stage 5, respectively, in adjusted model 2 (Table 3).

**Table 3:** Association of anemia with CKD. PNS, 2014–2015.

**Characteristics**	**Categories**	Crude model^*^	Adjusted model 1^**^	Adjusted model 2^***^
CKD stage	1	–	–	–
	2	0.74 (0.64–0.86)	0.68 (0.58–0.80)	0.70 (0.59–0.83)
	3a	0.93 (0.71–1.20)	0.73 (0.55–0.97)	0.65 (0.48–0.88)
	3b	2.82 (1.97–4.04)	2.11 (1.45–3.08)	1.93 (1.30–2.87)
	4	8.97 (3.62–22.22)	6.63 (2.63–16.73)	6.10 (2.40–15.53)
	5	5.87 (2.34–14.69)	5.10 (2.01–12.92)	4.45 (1.56–12.64)
Sex	Male	–	–	–
	Female		1.69 (1.45–1.96)	1.59 (1.35–1.88)
Age group	18–29 years	–	–	–
	30–44 years		1.10 (0.88–1.38)	1.14 (0.89–1.46)
	45–59 years		0.97 (0.76–1.24)	0.92 (0.70–1.11)
	60 years or more		1.55 (1.20–2.00)	1.18 (0.88–1.59)
Education	No education/incomplete elementary school	–	–	–
	Complete elementary school/incomplete high school		0.91 (0.74–1.13)	0.88 (0.70–1.11)
	Complete high school/incomplete higher education		0.92 (0.77–1.10)	0.87 (0.72–1.06)
	Complete higher education		0.70 (0.53–0.92)	0.65 (0.49–0.87)
Race	White	–	–	–
	Black		1.78 (1.41–2.25)	1.76 (1.36–2.26)
	Others (indigenous and yellow)		0.81 (0.43–1.52)	0.75 (0.38–1.52)
	Brown		1.19 (1.01–1.39)	1.20 (1.01–1.41)
Nutritional status	Low weight	–	–	–
	Eutrophy			0.66 (0.49–0.90)
	Overweight			0.49 (0.36–0.67)
Arterial hypertension				1.26 (1.04–1.52)
Diabetes mellitus				1.30 (1.00–1.69)
Cardiovascular disease				1.24 (0.91–1.69)
Smoking				0.71 (0.56–0.91)
Consumption excessive alcohol				1.13 (0.88–1.46)

^*^Association of anemia with the different stages of CKD. ^**^Association adjusted for sociodemographic variables (sex, age group, education, and race/color). ^***^Association adjusted for sociodemographic and clinical variables (sex, age group, education, race/color, arterial hypertension, diabetes mellitus, cardiovascular disease, smoking, nutritional status, and excessive alcohol consumption).

Furthermore, female individuals were more likely to have anemia than males after adjustments in models 1 and 2. Another factor that showed a difference was race, with black and brown individuals having, respectively, 1.76 and 1.20 times more likely to have anemia when compared to white individuals in adjusted model 2. Furthermore, the presence of AH and DM was associated with the presence of anemia (Table 3).

Higher education was a protective factor when compared to individuals with no education or who did not complete primary education (OR = 0.65; CI, 0.49–0.87). Regarding BMI, both overweight (OR = 0.49; CI, 0.36–0.67) and normal weight (OR = 0.66; CI, 0.49–0.90) showed protective effects against anemia compared to underweight. Furthermore, smoking was also a protective factor for anemia (OR = 0.71; CI, 0.56–0.91; Table 3).

## 4 Discussion

In this study, the presence of anemia was related to higher prevalences of AH (31%), DM (11.1%), CVD (6.4%) and CKD (15.1%). Furthermore, the groups most affected by anemia were women, aged between 30 and 44 years, of brown race/color, and with low education. Concerning the stages of CKD, the most advanced ones had a higher prevalence of anemia. When associating anemia with the different stages of CKD, greater chances of anemia were observed as the disease progressed. These findings remained significant after adjustments for sociodemographic and clinical variables.

A prevalence of anemia of 15.1% was found in individuals with CKD. Similarly, in a study carried out with the United States (USA) population, based on data from the National Health and Nutrition Examination Survey (NHANES)–a survey that evaluates the US population–from 2007 to 2010, a prevalence of anemia of 15.4% was demonstrated in individuals with CKD (10). Furthermore, in a cross-sectional cohort study conducted with the Japanese population, an even higher prevalence of anemia was found in individuals with CKD stage 4 (40.1%) and stage 5 (60.3%) (25).

This study also identified greater chances of developing anemia as CKD progresses. Corroborating this finding, a meta-analysis study in sub-Saharan African countries also found a relationship between anemia and CKD (26). This association is reinforced by the higher prevalence of anemia in the more advanced stages of this disease identified in the present study. In agreement, other studies have identified a higher prevalence of anemia according to the progression of CKD (9, 10, 13).

It is assumed that this association is due to the mechanism that patients with CKD have a deficiency in the production of erythropoietin, considering that it is synthesized by the kidneys (27). The progressive decline in endogenous erythropoietin (EPO) levels is traditionally recognized as a critical factor. EPO, a glycoprotein, is primarily produced by fibroblast-like peritubular interstitial cells in the kidneys. In individuals with Chronic Kidney Disease (CKD), EPO levels are inadequately low relative to the severity of anemia (28). Thus, according to the Guidelines of the Brazilian Society of Nephrology for managing anemia in chronic renal failure (29), these factors are responsible for the reduction in survival and quality of life of these individuals.

In this sense, it is important to highlight that, in the present study, individuals of black and brown race/color were, respectively, 76 and 20% more likely to have anemia than individuals of white race/color. This association can be explained by restricted access to health services in specific groups and diverse dietary patterns among ethnic groups (15). Similarly, other studies found evidence that sociodemographic and economic characteristics are related to a higher prevalence of anemia (30, 31). Furthermore, higher education proved to be a protective factor against anemia. This can be explained by the fact that, with greater education, the chances of employment and income increase, which allows greater access to information and the search for health services (32).

Another critical point is that women were 59% more likely to have anemia when compared to men. This can be explained by human physiology itself, as women experience blood loss during the menstrual cycle and, therefore, in pre-menopause, there is a greater risk of iron deficiency due to blood loss during menstruation (27). This information is consistent with the findings in this study, considering that the most affected groups were women aged 30–44 years. In other studies, a relationship was also found between the female sex and the presence of anemia (13, 26), reinforcing the data in the present study. Furthermore, women tend to seek health services more often, which can lead to greater reporting in these cases (33).

It is important to highlight that the association between anemia and the more advanced stages of CKD also remained after adjustment for clinical variables. Greater chances of anemia were found in individuals with DM and AH (34). Studies suggest that this association is multifactorial and can be explained by the presence of chronic diseases (such as CKD), diabetic nephropathy, older age, cardiovascular diseases, and low weight (13, 31). Regarding the association of DM and AH with CKD, these are the leading causes of this disease (1). This can be explained by the fact that CKD is generally present together with other comorbidities (35).

Regarding CVD, a higher prevalence was identified in individuals with anemia. This is because anemia can lead to poor functionality of existing cardiovascular lesions or even induce their appearance (36). Furthermore, CVD is associated with CKD due to long-term exposure to anemia and/or AH and inadequate treatment of these, which predisposes to the development of left ventricular hypertrophy (LVH) (37).

Our group's study demonstrated the importance of evaluating metabolic factors, such as AH, DM, and CVD, in individuals with CKD (38). As anemia has a strong relationship with CKD, these findings are also in line with the present study, as higher chances and prevalence of AH and DM and a higher prevalence of CVD were identified in individuals with anemia.

On the other hand, nutritional status and smoking were identified as protective factors for anemia. Regarding nutritional status, it was observed that eutrophic or overweight individuals had a lower chance of developing anemia compared to underweight individuals. Similarly, in a study conducted with women from Bangladesh, a higher risk of anemia was found when they were underweight and a lower risk of anemia in overweight/obese women compared to eutrophic women (39). The authors of this study explain that as the body mass index (BMI) increases, hemoglobin levels in the blood tend to increase while the risk of developing anemia decreases. On the other hand, another study demonstrated a higher prevalence of anemia in obese individuals compared to eutrophic women (40). Therefore, the information contained in the literature is still controversial, requiring further studies on the subject.

Regarding smoking, the observed association is presumed to be secondary and influenced by confounding factors, and can be explained by the following mechanism: during smoking, the released carbon monoxide binds to hemoglobin with high affinity, impairing oxygen binding. As a compensatory response, the body increases the production of erythrocytes, leading to a higher red blood cell count and a reduction in the erythrocyte sedimentation rate, which can distort the assessment of hemoglobin levels and make it difficult to identify anemia (41, 42). In this context, the association between tobacco use and anemia may reflect secondary changes in hematological parameters, rather than a truly protective effect, since smoking is considered an independent risk factor for CKD (43). Furthermore, a systematic review (44) demonstrated that smoking is a risk factor for both the progression of chronic kidney disease and anemia.

This study presents both strengths and limitations. Regarding limitations, the cross-sectional design of the study does not allow for the establishment of causal relationships between anemia and CKD or other variables analyzed. Furthermore, the study relied on self-reported clinical information, such as AH, DM, hypercholesterolemia and CVD, which may have affected the accuracy of the data due to potential recall or reporting biases. Due to logistical limitations, participant refusal, and difficulties in locating individuals, it was not possible to obtain all planned blood samples, which may have reduced the representativeness of the laboratory subsample in relation to the total study population. Finally, the study results may have been influenced by unmeasured confounding factors that were not included in the development of the analytical model. Therefore, future prospective cohort studies could confirm a causal relationship between anemia and CKD.

Regarding its strengths, the study adopts a carefully structured multi-stage probabilistic sampling strategy, which contributes to greater robustness of the results and reduces the influence of selection bias. The inclusion of laboratory data, such as hemoglobin levels and glomerular filtration rate, strengthens the validity of staging anemia and chronic kidney disease. Furthermore, the analysis considers several potential confounding factors through the use of stratified logistic regression models adjusted for demographic and clinical variables. The use of a stratified model also allows for examining the association between different stages of chronic kidney disease and the risk of anemia, enabling a more detailed understanding of how the risk of anemia progresses with disease progression. Finally, this study fills an important gap in the literature by investigating the prevalence of anemia in chronic kidney disease in the Brazilian population. By using a nationally representative laboratory subsample and standardized definitions for anemia and GFR, the results provide a benchmark for other middle-income countries with similar CKD and anemia burdens, supporting regional comparisons and public policy planning for early detection and management.

In conclusion, this study identified the variables related to the presence of anemia in the different stages of CKD. This suggests that patients in the more advanced stages of CKD have a greater chance of developing anemia and that there is an interrelationship with other comorbidities, such as AH and DM, in addition to sociodemographic characteristics, such as female sex, education, race/color, and nutritional status.

Therefore, it is essential to closely monitor the variables that indicate an increased likelihood of anemia in specific groups. To this end, the results of this study will serve as a reference for implementing and enhancing strategies aimed at ensuring the effective management of anemia in patients with CKD through public policies. Furthermore, it is anticipated that the findings will assist healthcare professionals in evaluating the prevalence of anemia and implementing appropriate clinical interventions. These may include health education initiatives for this population and more effective screening processes aimed at early detection. Recognizing the common risk factors associated with anemia will facilitate the identification of individuals at higher risk for its development.

## Data Availability

The datasets presented in this study can be found in online repositories. The names of the repository/repositories and accession number(s) can be found below: https://biblioteca.ibge.gov.br/visualizacao/livros/liv91110.pdf.

## References

[1] Kovesdy CP. Epidemiology of chronic kidney disease: an update 2022. Kidney Int Suppl. (2022) 12:7–11. doi: 10.1016/j.kisu.2021.11.003 35529086 PMC9073222

[2] Hill NR Fatoba ST Oke JL Hirst JA O'Callaghan CA Lasserson DS . Global prevalence of chronic kidney disease – a systematic review and meta-analysis. PLoS ONE. (2016) 11:1–18. doi: 10.1371/journal.pone.0158765 27383068 PMC4934905

[3] GBD Chronic Kidney Disease Collaboration. Global, regional, and national burden of chronic kidney disease, 1990–2017: a systematic analysis for the global burden of disease study 2017. Lancet. (2020) 395:709–33. doi: 10.1016/S0140-6736(19)32977-0 32061315 PMC7049905

[4] Foreman KJ Marquez N Dolgert A Fukutaki K Fullman N McGaughey M . Forecasting life expectancy, years of life lost, and all-cause and cause-specific mortality for 250 causes of death: reference and alternative scenarios for 2016–40 for 195 countries and territories. Lancet. (2018) 392:2052–90. doi: 10.1016/S0140-6736(18)31694-5 30340847 PMC6227505

[5] Brazilian Society of Nephrology. World Kidney Day 2013. São Paulo: SBN (2013).

[6] Quadros KAN Aquino JAd Vasconcelos FECd Guedes JVM Morais FAd Ribeiro FHR . Preventive and therapeutic approach to mineral and bone disorders in the early stages of chronic kidney disease: systematic review. Rev Eletron Acervo Saude. (2020) 12:e4067. doi: 10.25248/reas.e4067.2020

[7] Chinnappa S White E Lewis N Baldo O Tu YK Glorieux G . Early and asymptomatic cardiac dysfunction in chronic kidney disease. Nephrol Dial Transplant. (2018) 33:450–8. doi: 10.1093/ndt/gfx064 28525624

[8] Hanna RM Streja E Kalantar-Zadeh K. Burden of anemia in chronic kidney disease: beyond erythropoietin. Adv Ther. (2021) 38:52–75. doi: 10.1007/s12325-020-01524-6 33123967 PMC7854472

[9] Kidney Disease: Improving Global Outcomes (KDIGO) CKD Work Group. KDIGO clinical practice guideline for anemia in chronic kidney disease. Kidney Int Suppl. (2012) 2:279–335.

[10] Stauffer ME Fan T. Prevalence of anemia in chronic kidney disease in the United States. PLoS ONE. (2014) 9:1–4. doi: 10.1371/journal.pone.0084943 24392162 PMC3879360

[11] Gluba-Brzózka A Franczyc B Olszewski R Rysz J. The influence of inflammation on anemia in CKD patients. Int J Mol Sci. (2020) 21:725. doi: 10.3390/ijms21030725 31979104 PMC7036805

[12] Portolés J Martín L Broseta JJ Cases A. Anemia in chronic kidney disease: from pathophysiology and current treatments to future agents. Front Med. (2021) 8:642296. doi: 10.3389/fmed.2021.642296 PMC8032930 33842503

[13] Alemu B Techane T Dinegde NG Tsige Y. Prevalence of anemia and its associated factors among chronic kidney disease patients in public hospitals of Addis Ababa, Ethiopia. Int J Nephrol Renovasc Dis. (2021) 14:67–75. doi: 10.2147/IJNRD.S296995 33707966 PMC7943544

[14] Idris I Tohid H Muhammad NA A Rashid MR Mohd Ahad A Ali N . Anaemia among primary care patients with type 2 diabetes mellitus and chronic kidney disease: a multicenter cross-sectional study. BMJ Open. (2018) 8:1–9. doi: 10.1136/bmjopen-2018-025125 PMC6307578 30580276

[15] Nalado AM Mahlangu JN Waziri B Duarte R Paget G Olorunfemi G . Ethnic prevalence of anemia and predictors of anemia among chronic kidney disease patients at a tertiary hospital in Johannesburg, South Africa. Int J Nephrol Renovasc Dis. (2019) 18:19–32. doi: 10.2147/IJNRD.S179802 30858723 PMC6385786

[16] Alsalmani AA Alalawi NM Alsumri H Aljabri MK Alharami G Alweshahi R . Prevalence of anemia in primary care patients with type 2 diabetes mellitus and chronic kidney disease in Oman. J Family Community Med. (2023) 30:18–22. doi: 10.4103/jfcm.jfcm_226_22 36843861 PMC9954431

[17] Vlagopoulos PT Tighiouart H Weiner DE Griffith J Pettitt D Salem DN . Anemia as a risk factor for cardiovascular disease and all-cause mortality in diabetes: the impact of chronic kidney disease. J Am Soc Nephrol. (2005) 16:3403–10. doi: 10.1681/ASN.2005030226 16162813

[18] Santin F Canella D Borges C Lindholm B Avesani CM. Dietary patterns of patients with chronic kidney disease: the influence of treatment modality. Nutrients. (2019) 11:1–12. doi: 10.3390/nu11081920 31443269 PMC6723967

[19] Szwarcwald CL Malta DC Souza Júnior PRB Almeida WDS Damacena GN Pereira CA . National Health Survey laboratory tests: sampling methodology, data collection and analysis. Rev Bras Epidemiol.(2019) 22(suppl 2):1–9. doi: 10.1590/1980-549720190004.supl.2 31596375

[20] Instituto Brasileiro de Geografia e Estatística. National Health Survey 2013: Perception of Health Status, Lifestyles and Chronic Diseases. Rio de Janeiro: IBGE (2014).

[21] Kidney Disease: Improving Global Outcomes (KDIGO) CKD Work Group. KDIGO 2012 clinical practice guideline for the evaluation and management of chronic kidney disease. Kidney Int Suppl. (2013) 3:1–150.

[22] World Health Organization. Obesity: Preventing and Managing the Global Epidemic. Geneva: WHO (1998). 11234459

[23] Lipschitz DA. Screening for nutritional status in the elderly. Prime Care. (1994) 21:55–67. doi: 10.1016/S0095-4543(21)00452-8 8197257

[24] Centers for Disease Control and Prevention. Fact Sheets: Prevention Excessive Alcohol Use. Atlanta: CDC. (2024).

[25] Sofue T Nakagawa N Kanda E Nagasu H Matsushita K Nangaku M . Anemia and associated factors in chronic kidney disease patients: a cross-sectional study in a Brazilian cohort. PLoS ONE. (2020) 15:e0236132. doi: 10.1371/journal.pone.0236132 32687544 PMC7371174

[26] Taderegew MM Wondie A Terefe TF Tarekegn TT GebreEyesus FA Mengist ST . Anemia and its predictors among chronic kidney disease patients in Sub-Saharan African countries: a systematic review and meta-analysis. PLoS ONE. (2023) 18:1–17. doi: 10.1371/journal.pone.0280817 36730249 PMC9894480

[27] Raji YR Ajayi SO Akingbola TS Adebiyi AO Adedapo KS Salako BL. Assessment of iron deficiency anemia and its risk factors among adults with chronic kidney disease in a tertiary hospital in Nigeria. Postgrad Med. (2018) 25:197–203. doi: 10.4103/npmj.npmj_106_18 30588939

[28] Fujita S Wada T Tominari M. Management of anemia in chronic kidney disease: a comprehensive review of clinical guidelines and current practices. Front Med. (2021) 8:642296.

[29] Brazilian Society of Nephrology guidelines for managing anemia in chronic renal failure. Braz J Nephrol. (2000) 22(Suppl 5):1–3.

[30] Balarajan Y Ramakrishnan U Ozaltin E Shankar AH Subramanian SV. Anaemia in low-income and middle-income countries. Lancet. (2011) 378:2123–35. doi: 10.1016/S0140-6736(10)62304-5 21813172

[31] Mutter S Casey AE Zhen S Shi Z Mäkinen VP. Multivariable analysis of nutritional and socio-economic profiles show differences in incident anemia for Northern and Southern Jiangsu in China. Nutrient. (2017) 9:1–14. doi: 10.3390/nu9101153 PMC5691769 29065474

[32] Silva PA Justino TM Heitor RADS Santos FFD Barbosa AR Faria ACF . Association between the presence of iron deficiency anemia with socioeconomic variables and school performance. Med. (2018) 51:271–80. doi: 10.11606/issn.2176-7262.v51i4p271-280

[33] Pinheiro RS Viacava F Travassos C Brito AS. Gender, morbidity, access, and use of health services in Brazil. Science Saúde Colet. (2002) 7:687–707. doi: 10.1590/S1413-81232002000400007

[34] Chen CX Li YC Chan SL Chan KH. Anaemia and type 2 diabetes: implications from a retrospectively studied primary care case series. Hong Kong Med J. (2013) 19:214–21. doi: 10.12809/hkmj133814 23568938

[35] Corsonello A Fabietti P Formiga P Moreno-Gonzalez R Tap L Mattace-Raso F . Chronic kidney disease in the context of multimorbidity patterns: the role of physical performance. BMC Geriatrics. (2020) 20:1–12. doi: 10.1186/s12877-020-01696-4 PMC7532089 33008303

[36] Ammirati AL Canziani MEF. Risk factors for cardiovascular disease in patients with chronic kidney disease. Braz J Nephrol. (2009) 31:43–8.

[37] Fishbane S. Anemia and cardiovascular risk in the patient with kidney disease. Heart Fail Clin. (2008) 4:401–10. doi: 10.1016/j.hfc.2008.03.005 18760752

[38] Sousa LCM Silva NR Azeredo CM Rinaldi AEM Da Silva LS. Health-related patterns and chronic kidney disease in the Brazilian population: national health survey, 2019. Front Public Health. (2023) 11:1–8. doi: 10.3389/fpubh.2023.1090196 37089474 PMC10117670

[39] Kamruzzaman M. Is BMI associated with anemia and hemoglobin level of women and children in Bangladesh: a study with multiple statistical approaches. PLoS ONE. (2021) 16:1–18. doi: 10.1371/journal.pone.0259116 34710186 PMC8553127

[40] Cepeda-Lopez AC Osendarp SJ Melse-Boonstra A Aeberli I Gonzalez-Salazar F Feskens E . Sharply higher rates of iron deficiency in obesity Mexican women and children are predicted by obesity-related inflammation rather than by differences in diet iron intake. Am J Clin Nutr. (2011) 93:975–83. doi: 10.3945/ajcn.110.005439 21411619

[41] Nordenberg D Yip R Binkin NJ. The effect of cigarette smoking on hemoglobin levels and anemia screening. JAMA. (1990) 264:1556–9. doi: 10.1001/jama.1990.03450120068031 2395196

[42] Zanqueta EB Morais JF Yamaguchi MU. Hematological changes correlated with smoking. Int Meet Sci Prod. (2011) 1–6.

[43] Xia J Wang L Ma Z Zhong L Wang Y Gao Y . Cigarette smoking and chronic kidney disease in the general population: a systematic review and meta-analysis of prospective cohort studies. Nephrol Dial Transplant. (2017) 32:475–87. doi: 10.1093/ndt/gfw452 28339863

[44] Elihimas Júnior UF Elihimas HC Lemos VM Leão MdeA Sá MP França EE . Smoking as a risk factor for chronic kidney disease: systematic review. Braz J Nephrol. (2014) 36:519–28. doi: 10.5935/0101-2800.20140074 25517282

